# Dieta Intermitente na Remodelação Cardíaca Induzida pelo Exercício físico

**DOI:** 10.36660/abc.20200632

**Published:** 2020-08-19

**Authors:** Francis Lopes Pacagnelli, Andreo Fernando Aguiar, Letícia Estevam Engel, Antônio Cláudio Bongiovani, Mariana Janini Gomes

**Affiliations:** 1 Programa de pós-graduação em Ciência Animal Universidade do Oeste Paulista Presidente Prudente SP Brasil Programa de pós-graduação em Ciência Animal, Universidade do Oeste Paulista (Unoeste),Presidente Prudente, SP - Brasil; 2 Programa de pós-graduação em Ciências da Saúde Universidade do Oeste Paulista Presidente Prudente SP Brasil Programa de pós-graduação em Ciências da Saúde, Universidade do Oeste Paulista (Unoeste),Presidente Prudente, SP - Brasil; 3 Programa de Pós-Graduação Profissional em Exercício Físico na Promoção da Saúde Universidade Norte do Paraná Londrina PR Brasil Programa de Pós-Graduação Profissional em Exercício Físico na Promoção da Saúde, Universidade Norte do Paraná (UNOPAR), Londrina, PR - Brasil; 4 Departamento de Medicina Brigham and Women’s Hospital Harvard Medical School Boston MA USA Divisão de Medicina Cardiovascular, Departamento de Medicina, Brigham and Women’s Hospital, Harvard Medical School,Boston, MA - USA

**Keywords:** Dieta Intermitente, Coração, Exercício

O artigo intitulado: ‘Dieta Intermitente Atenua a Remodelação Cardíaca Causada pelo Exercício Físico’ apresenta informações relevantes sobre os efeitos do exercício físico (EF) na remodelação cardíaca, capacidade funcional, comportamento nutricional e metabolismo glicêmico. Os autores também analisaram a expressão de proteínas associadas a diferenciação celular e remodelação cardíaca, tais como a *extracellular signal-regulated kinase* (ERK) e *c-Jun N-terminal kinase* (JNK), a fim de determinar os possíveis mecanismos moleculares associados aos efeitos da dieta intermitente e EF. Os principais achados deste estudo foram que: 1) o EF isolado ou combinado a dieta intermitente aumentou a capacidade funcional, 2) a prescrição da dieta intermitente durante o programa de EF aumentou a tolerância glicêmica, 2) O EF isolado resultou na remodelação intersticial do miocárdio, e 3) a dieta intermitente foi capaz de atenuar a remodelação cardíaca induzida pelo EF.^1^

Vale ressaltar que este estudo utilizou um modelo experimental envolvendo ratos *Wistar* , a fim de simular uma dieta intermitente prescrita durante um programa de EF a longo prazo (12 semanas).^1^ A vantagem do modelo animal envolvendo aspectos nutricionais e físicos é a possibilidade de controlar as variáveis de confusão que podem afetar a validade interna da pesquisa, tais como o hábito alimentar, o estilo de vida, e a motivação intrínseca para o treino. Além disso, o modelo animal permite analisar os aspectos morfológicos, funcionais e moleculares do músculo cardíaco, possibilitando um estudo mais aprofundado dos mecanismos relacionados aos diversos tratamentos ou intervenções físicas e nutricionais. Desta forma, os resultados desse estudo são altamente consistentes e produzem informações relevantes para o entendimento e aprimoramento dos programas de prescrição de EF isolado ou associado a dieta intermitente. Esse estudo também aponta para uma nova direção de pesquisas sobre os efeitos do EF nos aspectos funcionais e morfológicos da remodelação cardíaca, e como a dieta intermitente pode modular esses efeitos.^1^

Estudos com a finalidade de compreender os efeitos benéficos do EF isolado ou combinado a estratégias nutricionais são fundamentais para a prevenção e tratamento de doenças cardiovasculares. Tal assunto tem recebido especial atenção nos últimos anos, uma vez que a Organização Mundial da Saúde revelou um número de aproximadamente 17,2 milhões de mortes por doenças cardiovasculares a cada ano, o que representa sério problema de saúde pública, além dos elevados custos financeiros com tratamentos e internações hospitalares.^2^ Assim, o controle dos fatores de risco cardiovascular e a preocupação com hábitos de vida saudáveis são essenciais para prevenção e limitação de danos cardiovasculares, destacando a importância dos estudos envolvendo condutas não farmacológicas, tais como o EF e a nutrição.

Neste contexto, a dieta intermitente e o EF são intervenções não farmacológicas que vêm sendo preconizadas há décadas para a promoção da saúde e o tratamento de doenças cardiovasculares. Entre os diversos benefícios dessas estratégias estão melhora da qualidade de vida, da composição corporal, dos níveis de colesterol e triglicérides, da resistência à insulina, prevenção da hipertensão e do desenvolvimento de aterosclerose.^3 , 4^ O impacto positivo da combinação de dieta intermitente e EF em diversas condições de saúde tem sido evidenciado em pesquisas experimentais e clínicas.^5 - 7^ Entretanto, a maioria dos estudos avaliaram os efeitos isolados do EF e da dieta intermitente, de modo que os efeitos da combinação destes tratamentos ainda não estão totalmente esclarecidos.

Estudo recente demonstrou o importante papel da dieta intermitente na redução de peso corporal e níveis de glicose sanguínea e de hemoglobina glicada, além de aumento da sensibilidade à insulina em camundongos obesos.^8^ Da mesma forma, o exercício aeróbio também tem demonstrado aumentar níveis proteicos musculares de GLUT 4 entre 20 a 70% em humanos e roedores, contribuindo para melhorar a sensibilidade à insulina e melhor controle glicêmico.^9^ Logo, é provável que a combinação do EF com a dieta intermitente poderia ser uma estratégia benéfica para potencializar a melhora do metabolismo glicêmico. Esta hipótese é suportada por um recente estudo que reportou maior eficiência do EF aeróbio combinado a restrição calórica moderada, do que o EF isolado, na melhoria da aptidão cardiorrespiratória, fadiga, incapacidade e controle glicêmico em idosos obesos.^10^ Similarmente, no artigo de Basilio et al.^1^ os autores verificaram melhora da capacidade funcional e do metabolismo glicêmico em ratos submetidos ao EF isolado ou combinado a dieta intermitente.^1^ Tais achados reforçam a ideia de que o EF isolado ou combinado a restrição calórica pode ser benéfico para promoção da saúde global.^1^

Estudos com a finalidade de investigar os mecanismos celulares e moleculares envolvidos na remodelação cardíaca em resposta ao EF e a restrição calórica são fundamentais para o entendimento e aplicabilidade destas estratégias em programas de prevenção e reabilitação da função cardiovascular. O estudo de Basilio et al.^1^ avaliaram o papel de algumas proteínas quinases ativadas por mitógeno (MAPK) na remodelação cardíaca induzida pelo EF e na dieta intermitente.^1^ A proteína p38 MAPK, apesar de não ter sido avaliada nesse estudo, é uma das mais importantes proteínas da via das MAPK, pois é ativada em resposta a estímulos como o EF. Essa proteína pode modular a função dos fibroblastos cardíacos e o turnover na matriz extracelular e da indução parácrina da hipertrofia dos cardiomiócitos.^11^ Portanto, futuros estudos são necessários para avaliar o papel da p38 MAPK na remodelação cardíaca induzida pelo EF e se há influência com a dieta intermitente.

A remodelação do miocárdio é regulada por uma combinação de respostas dos cardiomiócitos e de outros tipos celulares a várias vias mecanosensíveis, as quais podem modificar a expressão gênica e síntese proteica e assim promover modificações funcionais nessas células. Estímulos mecanoreguladores como o exercício físico atuam nos cardiomiócitos e fibroblastos e levam a alterações na expressão gênica e remodelação celular.^12^ Pesquisas relatam a importância das integrinas, angiotensina II, cálcio e TGFβ regulando a via proteínas quinases ativadas do mitógeno (MAPK) com ativação dos fibroblastos e aumento da fibrose cardíaca ^12 , 13^ .

Os efeitos da restrição calórica sobre a remodelação cardíaca têm sido muito investigados em modelos animais com alterações cardíacas. Foi demonstrado melhora da disfunção cardíaca e da reserva cronotrópica e, em relação a aspectos moleculares, melhora da inervação cardíaca simpática e níveis de receptores β-adrenérgicos em ratos com insuficiência cardíaca induzida por infarto do miocárdio submetidos a dieta intermitente.^14^ Outro estudo demonstrou que ciclos de jejum/realimentação promovem efeitos benéficos cardíacos e atenuam o dano miocárdico causado por restrição calórica em ratos espontaneamente hipertensos, contribuindo para reduzir o risco cardiovascular e os danos morfológicos. Além disso, o ciclo de jejum/realimentação promove leve melhora do trânsito do Ca^2^ e do sistema beta-adrenérgico.^15^ O estudo de Basilio et al.^1^ não teve como objetivo investigar os efeitos do EF isolado ou combinado a restrição calórica sobre marcadores de trânsito do Ca^2^ , mas apresenta novas possibilidades de estudos sobre as adaptações moleculares relacionadas a remodelação cardíaca induzida pelo EF associado a dieta intermitente..^1^ Ressaltamos a importância de testar os achados deste estudo em outras populações associadas a saúde e doenças, incluindo situações de sobrepeso, obesidade e diabetes, que cursam com alterações do metabolismo glicêmico.

Deste modo, vale novamente ressaltar que o artigo,^1^ apresenta valiosas informações sobre os efeitos do EF isolado ou combinado a dieta intermitente nos aspectos morfológicos e metabólicos envolvidos na remodelação cardíaca, contribuindo assim para o entendimento e aprimoramento dos programas de prevenção e reabilitação cardíaca.
