# Supplementary material for: Dieta Intermitente na Remodelação Cardíaca Induzida pelo Exercício físico
Source: Arq Bras Cardiol. 2020 Aug 19;115(2):194–6. [Article in Portuguese] doi: 10.36660/abc.20200632 (PMC8384287; doi:10.36660/abc.20200632)
Supplement: Translation

# Translation: Intermittent Diet in Exercise-Induced Cardiac Remodeling

**Authors:** Francis Lopes Pacagnelli, Andreo Fernando Aguiar, Letícia Estevam Engel, Antônio Cláudio Bongiovani, Mariana Janini Gomes

**Affiliations:** 1 Brazil Postgraduate program in Animal Science, University of Western São Paulo (Unoeste), Presidente Prudente, SP - Brazil; 2 Brazil Postgraduate program in Health Science, University of Western São Paulo (Unoeste), Presidente Prudente, SP - Brazil; 3 Brazil Postgraduate Program in Physical Exercise in Health Promotion, Northern University of Paraná, Londrina, PR - Brazil; 4 USA Division of Cardiovascular Medicine, Department of Medicine, Brigham and Women’s Hospital, Harvard Medical School, Boston, MA - USA

**Keywords:** Intermittent Diet, Heart, Exercise

The article entitled “Intermittent diet regulates physical exercise induced cardiac remodeling” presents relevant information concerning the effects of physical exercise (PE) on cardiac remodeling, functional capacity, nutritional behavior, and glycemic metabolism. The authors also analyzed the expression of proteins related to cellular differentiation and cardiac remodeling, such as extracellular signal-regulated kinase (ERK) and c-Jun N-terminal kinase (JNK), to determine the possible molecular mechanisms associated with the effects of intermittent diet and PE. The main findings were that: (1) PE increased functional capacity when isolated or combined with an intermittent diet, (2) prescription of an intermittent diet increased glycemic tolerance during PE programs, (3) intermittent diet was able to reduce cardiac remodeling induced by PE.^1^

It is worth highlighting that this study used an experimental model from *Wistar* rats to mimic an intermittent diet during a long-term PE program (12 weeks).^1^ The advantage of animal experiments involving nutritional and physical interventions is the possibility of controlling the confounding variables that can affect the internal validity of the study, such as food intake, lifestyle, and motivation for physical training. In addition, animal models allow for the analysis of morphological, functional, and molecular aspects of cardiac muscle, which enables a more in-depth study of the mechanisms related to the diverse treatments and physical and nutritional interventions. Therefore, the results of this study are highly consistent and relevant for the understanding and improvement of prescribed PE programs, whether when isolated or associated with intermittent diet. The study also reveals a new path for research involving the effects of PE on functional and morphological aspects of cardiac remodeling, and the role intermittent diet in modulating these effects.^1^

Researches aiming to understanding the beneficial effects of isolated PE or the combination of PE with nutritional strategies are fundamental for the prevention and treatment of cardiovascular diseases. This topic has received special attention in recent years, as the World Health Organization indicated approximately 17.2 million deaths from cardiovascular diseases each year, which indicates an important public health issue due to its high costs with treatment and hospital admissions.^2^ Risk factor control and the maintenance of healthy lifestyle habits are essential for the prevention and attenuation of cardiovascular complications, highlighting the relevance of studies focusing on PE and nutrition.

In this context, non-pharmacological interventions involving PE and intermittent diet have been advocated for decades to prevent and treat cardiovascular diseases. The benefits of these strategies include improved quality of life, body composition, and cholesterol and triglyceride levels, as well as the prevention of hypertension and atherosclerosis.^3 , 4^ The positive impacts of the combination of an intermittent diet and PE in several health conditions have been shown in experimental and clinical researches.^5 - 7^ However, most studies have evaluated the effects of PE and intermittent diet separately. Thus, the effects of a combination of these treatments are still not completely clear.

A recent study demonstrated the important role of intermittent diet on the reduction of body weight, blood glucose levels, and glycated hemoglobin levels, as well as increasing insulin sensitivity in obese mice.^8^ Similarly, aerobic exercise has also been shown to increase muscle expression of glucose transporter type 4 (GLUT-4) in 20 to 70%, both in humans and rodents. This contributes to improving insulin sensitivity and glycemic control.^9^ Therefore, it is likely that the combination of PE and intermittent diet could be a more powerful strategy to improve glycemic metabolism. This hypothesis is supported by a recent study that reported greater effects of PE combined with moderate caloric restriction than isolated PE on functional capacity, levels of fatigue and disability, and glycemic control in obese elderly individuals.^10^ Similarly, in the article by de Basilio et al.^1^ verified improved functional capacity and glycemic metabolism in rats that were submitted to isolated PE or combined with an intermittent diet. These findings reinforce the health benefits from isolated PE and its combination with a caloric-restricted.^1^

Studies aiming to investigate the cellular and molecular mechanisms involved in cardiac remodeling in response to PE and caloric restriction are fundamental to the understanding and application of these strategies in preventive programs and cardiovascular function rehabilitation. The study de Basilio et al.^1^ highlights the important evaluation of some mitogen-activated protein kinase (MAPK).^1^ Despite not being evaluated in this study, p38 MAPK is one of the most important proteins in the MAPK pathway due to its activity in response to stimuli like PE. It can modulate the function of cardiac fibroblasts, extracellular matrix turnover, and paracrine induction of cardiomyocyte hypertrophy.^11^ Therefore, future studies are needed to confirm the role of p38 MAPK in cardiac remodeling induced by PE and an influence of intermittent diet.

Myocardial remodeling is regulated through a combination of responses from cardiomyocytes, other cell types, and various mechanosensitive pathways, which can modify the genetic expression and protein synthesis, which cause the functional modifications in the cells.^12^ Mechanosensitive stimuli like PE affect cardiomyocytes and fibroblasts leading to alterations in genetic expression and cellular remodeling. Studies have shown the importance of integrins, angiotensin II, calcium, and transforming growth factor beta (TGF-β) regulating the mitogen-activated protein kinase (MAPK) pathway with fibroblasts activation and increased cardiac fibrosis.^12 , 13^

The effects of caloric restriction on cardiac remodeling have been extensively investigated in animal models with cardiac alterations. It has been demonstrated an improvement in cardiac dysfunction and chronotropic reserves, and regarding molecular aspects, an improvement in sympathetic cardiac innervation and levels of β-adrenergic receptors in rats with myocardial infarction-induced heart failure submitted to intermittent diet.^14^ Another study showed that fasting/refeeding cycles resulted in beneficial cardiac effects and reduced myocardial damage due to the caloric restriction in spontaneously hypertensive rats, contributing to reduced cardiovascular risks and morphological damage. In addition, fasting/ refeeding cycles led to slight improvements in the transit of Ca^2^ and the beta-adrenergic system.^15^ Although the study de Basilio et al.^1^ did not aim to investigate the effects of isolated PE or combined with caloric restriction diet on Ca^2^ transport markers, it shows possibilities for future researches on molecular adaptations related to cardiac remodeling induced by PE.^1^ We emphasize the importance of testing the findings of this study in other populations associated with health and disease, including groups with overweight, obese, and diabetic subjects such conditions are related to changes in glycemic metabolism.

In conclusion, it is worth highlighting that the article,^1^ demonstrates valuable information concerning the effects of isolated PE and the combination of PE with an intermittent diet on morphological and metabolic aspects involved in cardiac remodeling, such contributes to the understanding and enhancement of prevention programs and cardiac rehabilitation.
